# Case of idiopathic and complete appendiceal intussusception

**DOI:** 10.1111/ases.12442

**Published:** 2017-12-07

**Authors:** Ryoichi Tsukamoto, Kazuhiro Sakamoto, Kumpei Honjo, Koichiro Niwa, Kiichi Sugimoto, Shun Ishiyama, Hirohiko Kamiyama, Makoto Takahashi, Atsushi Okuzawa

**Affiliations:** ^1^ Department of Coloproctological Surgery, Faculty of Medicine Juntendo University Tokyo Japan

**Keywords:** Appendiceal intussusception, intestinal intussusception, laparoscopic surgery

## Abstract

Appendiceal intussusception is a rare disease in which the appendix invaginates into the cecum. It is often caused by organic diseases. The present case involved an appendiceal intussusception without an organic disease, and laparoscopic resection of part of the cecum was performed. Appendiceal intussusception has various causes, including malignant diseases. Therefore, diagnosis and selection of operative method are complex and could potentially lead to an excessively invasive option. By performing SILS with a multiuse single‐site port, we were able to provide an appropriate, non‐invasive treatment that had a good esthetic outcome.

## Introduction

In appendiceal intussusception, the appendix invaginates into the cecum 1. The prevalence of this disease has been reported to be 0.01% among patients who have undergone an appendectomy. Appendiceal intussusception is often caused by various organic diseases, but sometimes it occurs without organic diseases. Treatment of appendiceal intussusception differs significantly depending on whether the causative disease is malignant or benign. Therefore, preoperative diagnosis, including a histopathological examination, is important.

## Case Presentation

The patient was a 20‐year‐old woman. She visited the hospital with a primary complaint of lower abdominal pain with repeated remission and exacerbation that had lasted for 3 months.

**Figure 1:**
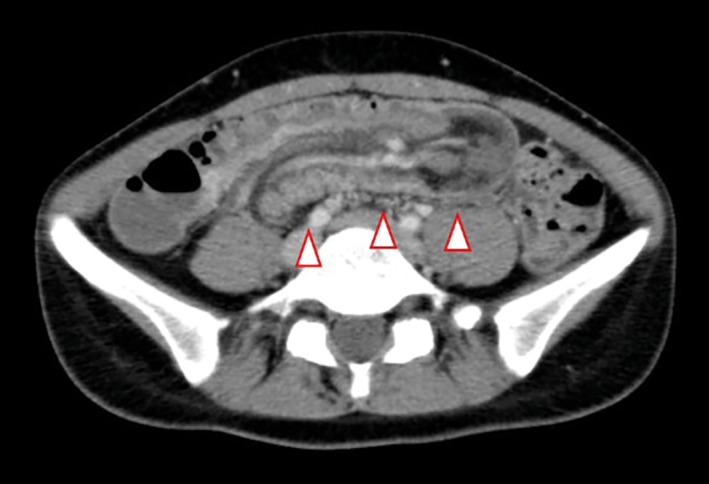
Transverse colon wall thickening and invagination on the oral side of the intestine were confirmed (arrowheads).

There was no abnormality in vital signs at the time of her initial visit. There was tenderness in the lower abdomen, and a neoplastic mass with mobility was palpable. Blood biochemistry confirmed that white blood count was 8700/μL and C‐reactive protein was 0.3 mg/dL, indicating mild inflammation. Abdominal CT confirmed invagination of the intestinal tract inside of the transverse colon, which led to a diagnosis of intestinal intussusception (Figure 1).

**Figure 2:**
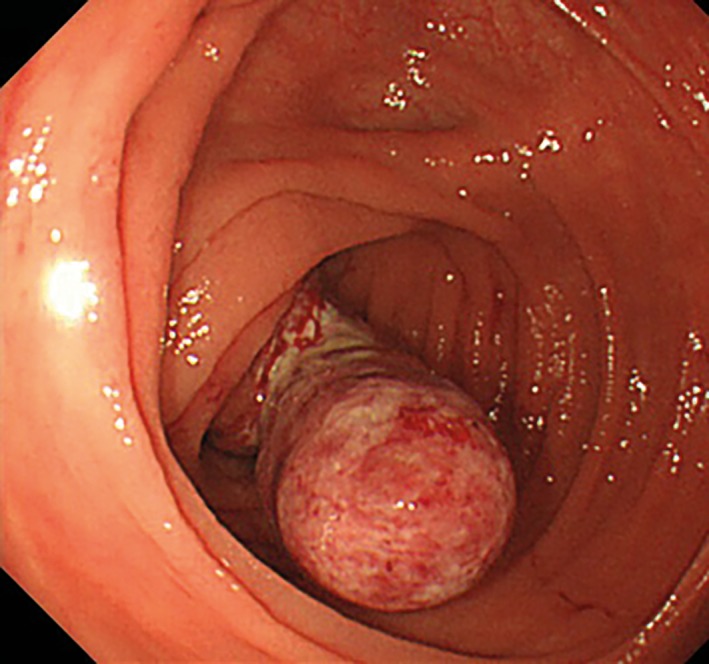
A protuberant lesion that was 30 mm in size was observed in the cecum.

The patient was hospitalized for emergency treatment, and a colonoscopic disinvagination was attempted without preparation. Invagination of a neoplastic lesion into the cecum was confirmed (Figure 2). A histopathological examination confirmed only inflammatory cells, but there was no clear finding of a neoplasm. However, the possibility of a malignant tumor could not be ignored as a cause of invagination. The patient was diagnosed with appendiceal neoplasm, and surgery was performed.

**Figure 3:**
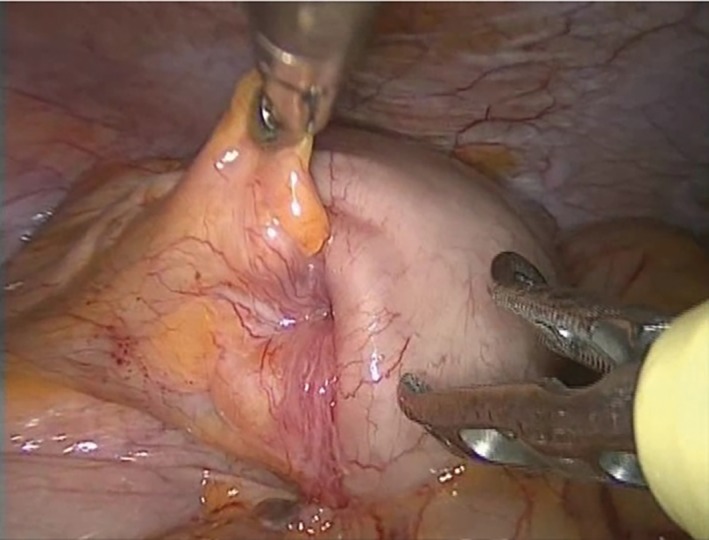
The appendix had inverted and invaginated into the cecum.

**Figure 4:**
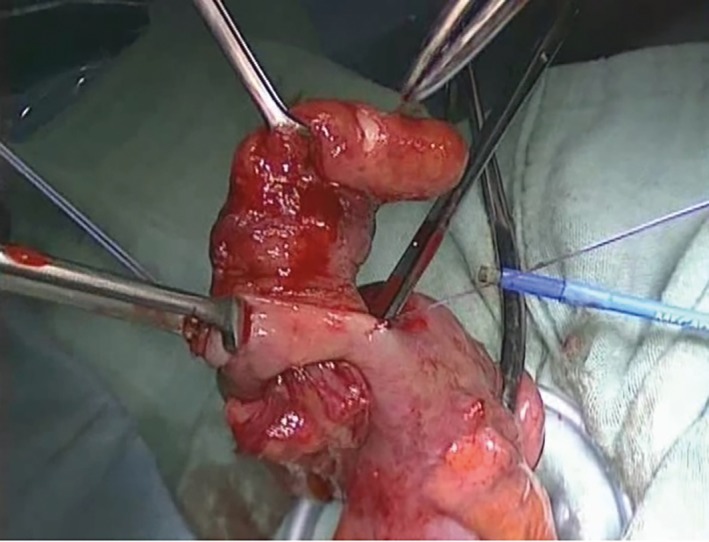
An incision was made in the cecum near the intussusception. The appendix was then retracted through the incision after the internal area was confirmed.

With the patient under general anesthesia, a 25‐mm midline incision was made in umbilical region. With a Lap‐Protector® (Hakko Medical, Tokyo, Japan) and EZ Access® (Hakko Medical), three 5‐mm ports were inserted in the same area to perform SILS. Intraperitoneal observation showed that the appendix had invaginated into the cecum, leading to a diagnosis of appendiceal intussusception (Figure 3). The tumor was probably resectable in the peritoneal cavity, but there was a risk of damaging the tumor. Therefore, the ileocecal region was extracted through the umbilical wound. With a small incision in the cecum, the intussuscepted appendix was retracted (Figure 4), and the cecum was partially resected with an automatic suturing device to keep the safe margin.

There were no malignant findings in the frozen section. Additional resection was deemed unnecessary, and the surgery was completed. In histopathological findings, the appendix was completely invaginated into the cecum. Because only inflammatory changes were confirmed in the appendiceal mucous membrane, there was no clear neoplastic component. The findings led to a diagnosis of idiopathic type V appendiceal intussusception.

The postoperative course was good, and the patient was discharged on the fifth day postoperatively.

## Discussion

Appendiceal intussusception is a rare disease. A case was first reported by McKidd *et al.* in 1858 2, and the prevalence of this disease has been reported to be 0.01% among patients who have undergone an appendectomy 1. Appendiceal intussusception has been reported to be more common in adults (76%) than in children (24%) 3, and it is twice as likely to be diagnosed in female adults (72%) than in female children 3. Digestive symptoms include abdominal pain, vomiting, and bloody bowel discharge 3. Reported pathological findings from 191 patients included endometriosis (33%), inflammatory change (19%), mucous cyst (19%), adenoma (11%), carcinoid (7%), and adenocarcinoma (6%) 3.

Appendiceal intussusception is classified based on the classification system by McSwain 4:

- Type I. The tip of the appendix forms the intussusceptum and is invaginated into the proximal appendix, which is the intussuscipiens.
- Type II. The invagination starts at some point along the length of the appendix. The intussuscipiens is the adjacent tissue.
- Type III. The invagination starts at the junction of the appendix and cecum. The cecum is the intussuscipiens.
- Type IV. This is a retrograde intussusception; the proximal appendix is invaginated into the distal appendix.
- Type V. The appendix is completely invaginated into the cecum and has progressed from type I–III.

The present case was classified as type V. It presented as only inflammatory changes without organic disease, and it was idiopathic.

It is difficult to identify the cause of appendiceal intussusception, and treatment policy should depend on the causative disease. However, according to one report, only 32% of cases had a causative disease that was determined preoperatively 1, Moreover, because intussusception forms a tumor mass, its cause may not be determined even during surgery. As such, it is difficult to diagnose whether it is benign or malignant.

If the cause is mucous cyst or adenoma, an appendectomy is adequate. However, carcinoid tumors and cancer require colectomy with lymph node dissection.

In the present case, the cecum was partially resected, and based on the results of a quick intraoperative histopathological examination, it was determined that additional resection was not needed.

There have been several reports on surgical treatment of appendix intussusception. The laparoscopic approach is advantageous because it allows observation within the entire abdominal cavity and minimizes physical contact with possible malignancies 5, 6, 7. In this case, appendectomy was the most minimally invasive procedure. Therefore, SILS was considered feasible as a means to minimize the invasion.

In conclusion, we presented a case of idiopathic and complete appendiceal intussusception. Appendiceal intussusception is a rare disease with diverse causes, which makes diagnosis and selection of the operative method difficult. Laparoscopic surgery offers a non‐invasive treatment that can be tailored to the individual, and it is considered to be an effective technique.

## Ethical statement

This study was conducted in accordance with the Declaration of Helsinki.
